# The influence of stem length on perioperative complications following cemented hip arthroplasty in metastatic femoral disease: A systematic review^☆^

**DOI:** 10.1016/j.jor.2025.07.029

**Published:** 2025-07-30

**Authors:** Joseph D. Giacalone, Jared Garfinkle, Surabhi Panda, Jason Abraham, Kassandra Parrales, Christopher Haydel

**Affiliations:** a Rowan-Virtua School of Osteopathic Medicine, Stratford, NJ, USA; b Virtua Reconstructive Orthopedics, Medford, NJ, USA

**Keywords:** Metastatic bone disease, Proximal femur, Cemented hip arthroplasty, Hip arthroplasty, Systematic review, Perioperative complications

## Abstract

**Introduction:**

Long stem cemented hip arthroplasty remains a common surgical treatment for metastatic disease to the proximal femur. However, long stems may increase the risk of cardiopulmonary complications due to embolic events. This study presents the first systematic review directly comparing perioperative complication rates between long and short/standard cemented femoral stems in this patient population.

**Methods:**

A systematic search of PubMed, EMBASE, Web of Science, and Cochrane Library was conducted in accordance with PRISMA guidelines. Studies were included if they reported on cemented hip arthroplasty for proximal femoral metastases, defined femoral stem length (short/standard: <250 mm vs. long: ≥250 mm), and described perioperative cardiopulmonary complications.

**Results:**

Seven studies met inclusion criteria, encompassing 379 femurs (160 short/standard stems and 219 long stems). Patients who received long-stem constructs had significantly higher rates of total perioperative cardiopulmonary complications (26.0 % vs. 3.1 %). Total complication rates were also higher in the long-stem group (28.8 % vs. 10.6 %). Only five cases (1.3 %) of new distal metastatic lesions were reported.

**Conclusion:**

Long cemented femoral stems are associated with higher perioperative complication rates than short or standard stems in patients undergoing hip arthroplasty for proximal femoral metastases. Given the low observed incidence of new distal lesions, the rationale for routinely using long stems warrants reconsideration. Future prospective studies should adopt standardized definitions for cardiopulmonary complications and stem length, report BCIS using validated criteria, and evaluate the true incidence of new distal metastases to guide surgical decision-making in this high-risk population.

## Highlights of the Paper

• First systematic review to compare complication rates between differing stem lengths in proximal femoral metastases.
• Long cemented stems were associated with higher rates of cardiopulmonary complications compared to shorter length stems.
• Findings may guide surgical decision-making by balancing fixation goals with perioperative risk profiles.

## 1 Introduction

Advancements in primary cancer treatment have led to increased life expectancy, however, this progress has resulted in subsequent rise in the incidence of metastatic bone disease and concomitant skeletal-related complications.^1^ Among these complications are cancer-induced bone pain, cachexia, and pathologic fractures. Approximately 68 % of all patients with skeletal metastasis experience pain^2^, while 2–28 % encounter pathologic fractures, where the variation is largely dependent on the oncologic subtype.^3^

Surgical interventions for pathologic fractures of the proximal femur resulting from bone metastasis traditionally include endoprosthetic reconstruction, plate osteosynthesis, and intramedullary nail fracture fixation.^4^, ^5^, ^6^, ^7^, ^8^ Endoprosthetic reconstruction -specifically long stem cemented hip arthroplasty (LSCHA) - is utilized in this patient population as it offers mechanical fixation to a significant amount of more distal femur, helping to prophylactically protect against recurrence or future metastases that could lead to fracture.^9^, ^10^, ^11^

However, there is ongoing debate in the literature regarding the necessity of this treatment. While some studies highlight the procedures’ efficacy,^12^, ^13^, ^14^ others challenge its use, pointing to increased complication rates and disputing the proposed benefits, especially in light of the low incidence of distal metastases and the generally poor survival rates in this patient population.^9^^,^^15^

In terms of operative complications, it has been suggested that the extended length of the prosthesis increases intramedullary pressure, which may lead to a higher embolic load^16^**;** a risk further exacerbated by the permeative, highly vascularized and hypercoagulable qualities of metastatic bone. Consequently, LSCHA has been associated with an increased risk of intraoperative bone cement implantation syndrome (BCIS), which can present with cement-related complications such as oxygen desaturation, hypotension, embolization, arrhythmias, cardiac arrest, and, in severe cases, death.^16^, ^17^, ^18^, ^19^, ^20^, ^21^, ^22^

Given this discourse, our study aimed to conduct a systematic review comparing rates of subsequent cardiopulmonary complications, distal metastasis, and survival, between cemented long and short femoral stems in patients with proximal femoral metastases.

## 2 Methods

A systematic search of the literature was conducted across four databases in June of 2025: PubMed, EMBASE, Web of Science, and the Cochrane Library. The search was performed in accordance with the Preferred Reporting Items for Systematic Reviews and Meta-Analyses (PRISMA) guidelines using a uniform Boolean search string. Keywords including “hip arthroplasty,” “metastatic disease,” “proximal femur,” “perioperative complications,” and “prosthesis stem length” were used to search each database. Search terms were required to appear in the title, abstract, or keywords of eligible studies. Logical operators (AND and OR) were applied to refine the search strategy and link related terms, including variations such as “hip replacement,” “bone metastases,” “femoral,” “cardiopulmonary complications,” and “cemented stem,” to ensure comprehensive retrieval of relevant literature.

Studies were included if they involved patients undergoing cemented hip arthroplasty for metastatic disease of the proximal femur and provided clear definitions of femoral stem length that could be categorized as short/standard (<250 mm) or long (≥250 mm). To be eligible, studies were also required to report on perioperative cardiopulmonary complications, including events such as hypotension, desaturation, embolism, cardiac arrest, and/or bone cement implantation syndrome (BCIS). Studies were excluded if they involved patients with periacetabular disease, failed to use or specify the use of cement, did not clearly define stem length categories, did not report cardiopulmonary complications, lacked data that could be stratified into short/standard or long stem groups, or were published as case reports.

For each included study (n = 7), data was collected using a single data extraction sheet to ensure consistency, and eventually pooled across all participants. Patient demographic variables included the patient gender, and reported age (mean or median). Oncologic variables included the primary carcinoma type, presence of impending or pathologic fracture, Mirels score (when available), and history of prior femoral lesions. Surgical details focused on femoral stem length, which was categorized as short/standard (<250 mm) or long (≥250 mm).

Perioperative outcomes were also documented, and included intraoperative and postoperative: hypotension, oxygen desaturation, death, respiratory distress, cardiac arrest, pulmonary embolism, pneumonia, aseptic loosening, periprosthetic fractures, infections, and reoperations. Additional measured complications included total and percent rates of cardiovascular complications and distal femoral disease progression. When reported, Musculoskeletal Tumor Society (MSTS) scores, patient survival rates, and mean or median follow-up durations were also extracted. All outcome data was recorded as reported in the original studies, without standardizing definitions beyond the categorization of stem length.

The quality of included studies was independently assessed by one reviewer (JG) using the Methodological Index for Non-Randomized Studies (MINORS) criteria. This validated tool is widely used for evaluating non-randomized surgical studies, which comprised the majority of studies included in our review. In particular, of the seven studies assessed, six were non-randomized, while one study (Singh et al.;^24^) was a randomized controlled trial and one (Abdelmonem et al.;^9^) employed a quasi-randomized design. While a more specific risk-of-bias tool, such as the Cochrane Risk of Bias 2 tool, could have been used for the randomized trial, applying a single instrument across all studies permitted a more uniform comparison. The MINORS criteria consists of 12 items: 8 for non-comparative studies and 4 additional items for comparative designs. Each item is scored as 0 (not reported), 1 (inadequately reported), or 2 (adequately reported), which results in a maximum score of 16 for non-comparative studies and 24 for comparative studies. For interpretive purposes, MINORS scores were categorized as high (20–24 for comparative studies; 13–16 for non-comparative), moderate (13–19 or 9–12), or low quality (≤12 or ≤ 8), consistent with prior literature using this tool.

Due to heterogeneity in study design, outcome definitions, and quality among the included studies, we did not perform a formal meta-analysis. As such, results were synthesized descriptively. We extracted complication rates related to both perioperative cardiopulmonary outcomes and implant-related events, stratified by femoral stem length (short/standard vs. long). When available, individual complication counts were pooled, and total complication rates were calculated as simple proportions. Although odds ratios, confidence intervals, and p-values were generated for exploratory purposes, no statistical adjustment for confounding or inter-study variability was performed. These values should therefore be interpreted with caution and not as evidence of causality. This approach enabled us to summarize clinical trends while acknowledging the methodological limitations of the underlying data.

## 3 Results

**Fig. 1:**
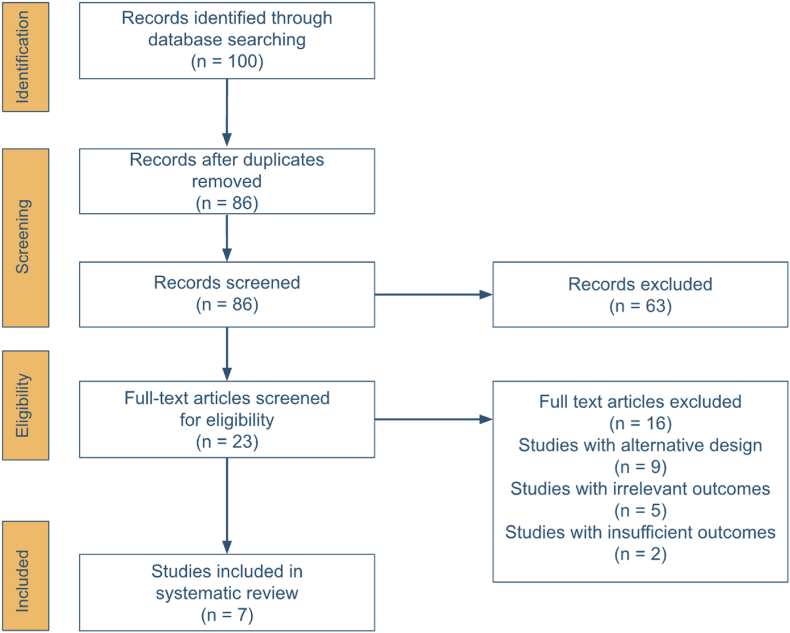
PRISMA flow chart summarizing the selection process for studies included in our systematic review.

The initial search yielded 100 studies. After removal of 14 duplicates and manual screening for eligibility, 7 articles were found to meet inclusion criteria^9^^,^^12^, ^13^, ^14^, ^15^^,^^23^^,^^24^ (Fig. 1). Although the study by Herrenbruck et al.^18^ is frequently cited in the literature on cardiopulmonary complications from LSCHA, it was excluded because it encompassed a wide range of implant lengths (155–300 mm; mean 232 ± 40.5 mm), which precluded consistent categorization and comparison between short/standard and long stems according to our study criteria.

Based on our quality assessment using the MINORS tool, two of the seven included studies achieved a high methodological score (≥22/24): Abdelmonem et al. (22/24) and Singh et al. (20/24). The remaining five studies demonstrated moderate quality, with scores of 16 or 17 out of 24 for comparative studies and 9 or 10 out of 16 for all non-comparative studies. Among the 12 criteria, the most consistently adequately reported were a clearly stated aim (criterion 1), inclusion of consecutive patients (criterion 2), and endpoints appropriate to the aim of the study (criterion 4); each of which was adequately reported in all 7 studies. In contrast, prospective collection of data (criterion 3) and prospective calculation of study size (criterion 8) were the least reported either adequately or inadequately, with 5 out of 7 studies failing to report criterion 3 and 6 out of 7 failing to report criterion 8 (Table 1).

**Table 1:** Quality assessment of included studies using the Methodological Index for Non-Randomized Studies (MINORS) criteria. Scores for each criterion are presented by study, with total scores shown in the final column. For comparative studies, the maximum possible score is 24; for non-comparative studies, the maximum is 16. The bottom rows summarize the number of studies in which each criterion was not reported (score = 0), was reported inadequately (score = 1), or adequately (score = 2). A dash (−) indicates that the criterion was not applicable due to the study's non-comparative design.

Study	1	2	3	4	5	6	7	8	9	10	11	12	Total Score
Xing et al. (15)	2	2	0	2	1	1	2	0	2	1	1	2	16/24
Abdelmonem et al. (9)	2	2	2	2	1	1	2	2	2	2	2	2	22/24
Price et al. (13)	2	2	0	2	1	1	1	0	–	–	–	–	9/16
Randall et al. (12)	2	2	0	2	1	1	1	0	–	–	–	–	9/16
Peterson et al. (14)	2	2	0	2	1	1	2	0	–	–	–	–	10/16
Naik & Lietman (22)	2	2	0	2	1	1	1	0	2	2	2	2	17/24
Singh et al. (24)	2	2	2	2	2	1	0	0	2	2	1	2	20/24
Not reported	0/7	0/7	5/7	0/7	0/7	0/7	1/7	6/7	0/4	0/4	0/4	0/4	
Inadequate	0/7	0/7	0/7	0/7	6/7	7/7	3/7	0/7	0/4	1/4	2/4	0/4	
Adequate	7/7	7/7	2/7	7/7	1/7	0/7	3/7	1/7	4/4	3/4	2/4	4/4	

A total of 379 femurs were included in the analysis, with 160 (42 %) receiving short/standard cemented femoral stems and 219 (58 %) receiving long cemented femoral stems. The cohort consisted of 161 males (43 %) and 212 females (57 %), with a mean age of 59.4 years. The most common primary malignancy was breast cancer (n = 117, 31.4 %). Additional histologic subtypes included adenocarcinoma of unknown origin (n = 9, 2.4 %) and others such as thyroid carcinoma, melanoma, and hepatocellular carcinoma (n = 68, 18.2 %). Disease progression was observed in 13 cases (3.4 %), while new distal lesions developed in 5 cases (1.3 %) (Table 2).

**Table 2:** Summary on patient demographics, tumor histology and characteristics.

Parameters	Totals	Percentage
**Gender**		
Male	161	43.2 %
Female	212	56.8 %
**Total patients**	373	
**Mean Age (years)**	59.4	
**Total femurs**	379	
Short Stems (<250 mm)	160	42.2 %
Long Stems (≥250 mm)	219	57.8 %
**Histologic Subtype**		
Breast	117	31.4 %
Renal	58	15.5 %
Lung	47	12.6 %
Multiple Myeloma	41	11.0 %
Prostate	29	7.8 %
Colon	10	2.7%
Adenocarcinoma (Unknown Origin)	9	2.4 %
Other	68	18.2 %
**Disease Progression**		
Short Stems (<250 mm)	5	3.1 %
Long Stems (≥250 mm)	8	3.7 %
Total Disease Progression	13	3.4 %
**New Distal Lesions**		
Short Stems (<250 mm)	3	1.9 %
Long Stems (≥250 mm)	2	0.9 %
Total New Distal Lesions	5	1.3 %

Patients who underwent long-stem arthroplasty experienced statistically significantly higher rates of intraoperative and/or postoperative cardiopulmonary complications compared to those receiving short stems (26.0 % vs. 3.1 %, OR = 0.09 [95 % CI: 0.04–0.23], p < 0.0001). Among individual complications, oxygen desaturation occurred significantly more often in the long-stem cohort (3.2 % vs. 0.0 %, OR = 0.09 [95 % CI: 0.01–1.56], p = 0.02). Hypotension was reported in 15.5 % of long-stem cases but was not reported for the short/standard group, precluding statistical comparison. Cardiac arrest (OR = 0.15 [95 % CI: 0.01–2.79], p = 0.14), death (OR = 0.27 [95 % CI: 0.01–5.11], p = 0.51), pulmonary embolism (OR = 0.45 [95 % CI: 0.05–4.39], p = 0.64), and postoperative pneumonia (OR = 0.78 [95 % CI: 0.22–2.70], p = 0.77) occurred at similar rates between groups, with differences not found to be statistically significant (Table 3).

**Table 3:** Comparison of intraoperative and postoperative cardiovascular complications between short/standard and long cemented femoral stems. Odds ratios (OR) with 95 % confidence intervals (CI) were calculated using Fisher's exact test. Odds ratio reflects odds of outcome in the short/standard stem group, with the long stem group as the reference. The Haldane-Anscombe correction was applied to zero-event cases to allow for odds ratio estimation. P-values <0.05 were considered statistically significant and are marked with an asterisk (∗*).* A dash (−) indicates that the outcome was not mentioned.

Complications (Intraoperative/Postoperative)	Short/Standard Stem (n = 160)	Long Stem (n = 219)	Odds Ratio (95 % CI)	P-value
Hypotension	–	34 (15.5 %)	–	–
Oxygen Desaturation	0 (0.0 %)	7 (3.2 %)	0.09 (0.01, 1.56)	0.02∗
Cardiac Arrest	0 (0.0 %)	4 (1.8 %)	0.15 (0.01, 2.79)	0.14
Pulmonary Embolism	1 (0.6 %)	3 (1.4 %)	0.45 (0.05, 4.39)	0.64
Death	0 (0.0 %)	2 (0.9 %)	0.27 (0.01, 5.11)	0.51
Pneumonia	4 (2.5 %)	7 (3.2 %)	0.78 (0.22, 2.70)	0.77
Total Cardiopulmonary Complications	5 (3.1 %)	57 (26.0 %)	0.09 (0.04, 0.23)	<0.0001∗

In terms of implant-related complications, aseptic loosening was more frequently observed in patients with short stems (OR = 9.76 [95 % CI: 0.60–190.20], p = 0.07). Periprosthetic fractures (OR = 0.34 [95 % CI: 0.04–3.05], p = 0.40) and re-operations (OR = 2.09 [95 % CI: 0.58–7.55], p = 0.33) were comparable between groups. Additionally, infection rates remained low in both cohorts (2 cases each, OR = 1.37 [95 % CI: 0.19–9.85], p = 1.00). Overall complication rates, calculated as the sum of total cardiovascular complications, infections, aseptic loosening, and periprosthetic fractures, were significantly higher in the long-stem group, with a total complication rate of 28.8 % compared to 10.6 % in the short-stem cohort (OR = 0.29 [95 % CI: 0.16–0.53], p < 0.0001) (Table 4). A detailed breakdown of pooled data by study source is provided in Supplementary Table 1.

**Table 4:** Comparison of intraoperative and postoperative implant-related complications between short/standard and long cemented femoral stems.“Total complications” represents the combined incidence of cardiovascular complications, infections, aseptic loosening, and periprosthetic fractures. Odds ratios (OR) with 95 % confidence intervals (CI) were calculated using Fisher's exact test. Odds ratio reflects odds of outcome in the short/standard stem group, with the long stem group as the reference. The Haldane-Anscombe correction was applied to zero-event cases to allow for odds ratio estimation. P-values <0.05 were considered statistically significant and are marked with an asterisk (∗).

Complications	Short/Standard Stem (n = 160)	Long Stem (n = 219)	Odds Ratio (95 % CI)	P-value (Fisher's Exact Test)
Infection	2 (1.3 %)	2 (0.9 %)	1.37 (0.19, 9.85)	1.00
Aseptic Loosening	3 (1.9 %)	0 (0.0 %)	9.76 (0.50, 190.20)	0.07
Periprosthetic Fracture	1 (0.6 %)	4 (1.8 %)	0.34 (0.04, 3.05)	0.40
Reoperations	6 (3.8 %)	4 (1.8 %)	2.09 (0.58, 7.55)	0.33
Total Complications	17 (10.6 %)	63 (28.8 %)	0.29 (0.16, 0.53)	<0.0001∗

Mirels scores were reported in only two studies: Price et al. (mean 10.5) and Randall et al. (mean 10.2). MSTS scores were provided by Abdelmonem et al. at 6 months (mean 23.3 for the standard stem group and 21.0 for the long stem group) and by Peterson et al., which reported a median MSTS score of 21 at the most recent follow-up. Survival data were inconsistently reported across five of the seven studies, with formats ranging from percentage survival at fixed intervals (Xing et al.), to average survival in months (Price et al., Abdelmonem et al., Randall et al.), and median survival (Peterson et al.). Mean or median follow-up duration was only explicitly reported in three studies: Xing et al., Abdelmonem et al., and Peterson et al. Due to these inconsistencies in reporting, we were unable to systematically compare these variables or draw conclusions regarding long-term outcomes.

## 4 Discussion

In the management of proximal femoral metastasis, LSCHA stands out as a surgical intervention, permitting sufficient access to the lesion for removal, while also providing stable fixation and protection against local recurrence and potential future distal disease. However, concerns persist regarding the association between LSCHA and significant cardiopulmonary complications, including bone cement implantation syndrome (BCIS). Several studies have reported embolic-related events, such as intraoperative hypotension, hypoxia, and cardiac arrest occurring in this patient cohort.^16^, ^17^, ^18^, ^19^, ^20^, ^21^, ^22^ Despite these risks, no prior study has conducted a systematic review directly comparing the incidence of such complications between long and short/standard cemented femoral stems in this patient population. Our study aimed to address this gap by examining rates of hypotension, desaturation, cardiac arrest, and other cardiopulmonary complications across existing literature. We therefore conducted a systematic review and pooled analysis of available orthopaedic literature, and found that long cemented femoral stems were associated with significantly higher rates of perioperative cardiopulmonary complications - namely oxygen desaturation - compared to shorter stems in patients undergoing hip arthroplasty for metastatic femoral disease.

There were several limitations included in the present study. First, due to significant heterogeneity in how perioperative complications were defined and reported across studies, we decided against performing a formal meta-analysis, and instead conducted a pooled descriptive analysis. These pooled results should be interpreted with caution, as they are derived from heterogeneous sources and are not adjusted for confounding variables. Although we reported p-values and confidence intervals for exploratory comparisons between groups, these statistical tests were applied only to summarize differences in pooled data and do not reflect patient-level analyses. As such, the statistical significance of these findings should not be over-interpreted and the results should not be viewed as confirmatory. In addition, no study included in our analysis included BCIS as a complication using a standard criteria, such as Donaldson's proposed classification.^16^ Second, the inability to isolate femoral stem length as the sole contributing factor limits our conclusions; while an association with cardiopulmonary complications was observed, it is likely that stem length interacts with cementation itself^25^ and other risk factors - including advanced age and concurrent lung metastases^19^ - to influence overall risk. Third, the included studies varied widely in surgical technique, implant type, and surgeon experience, contributing to additional heterogeneity.

Future randomized control trials or high quality prospective studies are required to make more statistically meaningful comparisons between the cardiopulmonary complication rates observed in long vs short cemented stems. These studies should aim to adopt consistent definitions for perioperative cardiopulmonary complications using established criteria in the literature, or alternatively, explicitly include BCIS as a reportable outcome. Additionally, an effort to establish a universally accepted classification of short, standard, and long femoral stem lengths would serve to clarify future literature surrounding the topic. Such standardization would allow for higher-quality comparisons across studies, enabling future meta-analyses to better assess complication profiles in these patients. Further research is warranted to determine the true incidence of new distal metastatic lesions in this patient population, as this may serve to either justify, or challenge, the routine use of long-stem constructs in proximal femoral metastases.

In our pooled analysis of 379 femurs undergoing cemented hip arthroplasty for proximal femoral metastases, it was found that those treated with long-stem constructs had a markedly higher incidence of total perioperative cardiopulmonary complications when compared to those with short or standard-length stems (26.0 % vs. 3.1 %). Perioperative oxygen desaturation was the only individual complication that occurred disproportionately more often in the long-stem group, with rates of 3.2 % compared to 0.0 % (Table 3). While this trend aligns with previously published concerns surrounding the physiologic impact of long cemented stems, our findings are among the first to quantify this risk across multiple studies using a stem length-based comparison. These results suggest that while long-stem constructs may offer extended diaphyseal fixation and theoretical protection against future lesions, they also carry a greater perioperative risk profile-especially in patients with additional risk factors.

In addition to cardiopulmonary events, we also examined implant-related complications. While individual complications such as infection, aseptic loosening, periprosthetic fracture, and reoperation did not differ significantly between groups, the total complication rate was significantly higher in the long-stem group compared to the short/standard-stem group (28.8 % vs. 10.6 %; Table 4). In a recent multi-center study of 337 patients by Lawrenz et al., they reported low cumulative rates of femoral stem revision and complication at 2 years in both cemented and uncemented treatment groups. Notably, cemented stems demonstrated a 1.5 % revision rate, a stem complication rate of 5.2 %, and a 2 % incidence of periprosthetic fracture.^26^

Interestingly, despite the theoretical benefit of spanning a greater length of the femur against new future distal metastases, we found a very low incidence of new distal lesions irrespective of stem length (1.3 %; Table 2). Notably, only two of the seven studies included in our review - those by Xing et al. and Naik and Leitman - explicitly reported on the occurrence of new distal lesions, suggesting that this outcome was underreported in the studies included in our review. However, existing literature suggests that the development of new femoral metastases following treatment of isolated lesions is uncommon.^10^^,^^15^^,^^27^ As such, the rationale for using long-stem constructs solely to prevent future distal disease remains largely unsubstantiated and warrants further prospective investigation.

Given our findings, it is worth discussing alternative treatment options for patients with metastatic lesions to the proximal femur. A lesion-specific and patient-tailored approach is generally recommended either with endoprosthetic reconstruction (EPR) or internal fixation (IF) with an intramedullary nail (IMN) or osteosynthesis with a plate and screw construct. EPR is typically favored in patients with lesions involving the femoral head, neck, or extensive peritrochanteric bone destruction, particularly when there is significant bone loss, a high risk of nonunion, or failure of prior IF.^28^ Proximal femur replacements have also shown to be an effective surgical option for metastatic lesions to the proximal femur.^29^ Conversely, IF may still be appropriate for subtrochanteric or diaphyseal lesions, particularly when bone stock is preserved and immediate weight bearing is prioritized.^28^^,^^30^ In such cases, the use of a long IMN with cement augmentation and interlocking screws placed into the femoral head, neck, and suprapatellar region is recommended.^28^^,^^30^^,^^31^ Plate-and-screw constructs, while an option in certain distal metaphyseal or epiphyseal lesions, are generally less favored in the proximal femur due to their association with higher complication and failure rates.^32^ Iljazi et al. conducted a recent systematic review of 34 studies and concluded that EPR is the preferred surgical strategy in patients with anticipated survival beyond six months, poor bone quality, or extensive local destruction. Although their review did not quantify perioperative cardiopulmonary complication rates, they reported lower revision and implant removal rates for EPR compared to IF, reinforcing the long-term durability advantage of prosthetic reconstruction in appropriately selected patients.^33^

Considering EPR is more commonly favored in patients with proximal femoral metastases, we believe it is important for future research to focus on optimizing the use of EPR, such as evaluating long versus standard stem constructs, in order to minimize cardiopulmonary risk in a treatment modality already associated with strong implant survival and low revision rates. While the general consensus in the current literature, along with the findings of our study, suggest an association between longer stem length and increased risk of perioperative cardiopulmonary complications, there is a paucity of high-quality prospective studies addressing this topic. The same applies to studies comparing these complications in patients undergoing cemented versus uncemented arthroplasty for proximal femoral metastases. It is worth noting that while cemented fixation remains a reliable option in this patient population, uncemented stems may also be appropriate in carefully selected patients; particularly those under 65 years old with good bone quality and sufficient remaining bone following curettage of macroscopic disease.^26^

For surgeons that prefer to utilize cemented hip arthroplasties, a multitude of adjustments to surgical and cementation techniques have been recommended to both improve outcomes and mitigate the risk of embolization events. These include thorough irrigation/pulse-lavage and drying/suctioning of the intramedullary canal, placing a cement-restrictor, vacuum cement mixing, retrograde cement introduction using a long-nozzle cement gun, and slow and controlled insertion of the femoral component into the medullary canal without pressurization.^34^, ^35^, ^36^

Pulse lavage and adequate suctioning of the femoral canal can mechanically remove tissue debris such as fat, blood and bone marrow from surface interstices and bony microstructures.^34^ As such, these techniques are speculated to promote interdigitation at the cement-bone interface and decrease the risk of embolization and BCIS.^34^^,^^35^ Vacuum cement mixing reduces cement porosity, thereby mitigating the formation of air microbubbles that could compromise cement integrity and potentially lead to the extrusion of gaseous microemboli.^34^

In addition to these methods, there has been extensive discussion regarding bone cement viscosity. Bone cement with exceedingly low consistency results in intercalation of the cement with residual bone marrow, fat, and debris, while exceedingly high consistency cement mitigates interdigitation at the cement-bone interface and may impede stem implantation.^34^^,^^35^ Results in the literature pertaining to the optimal viscosity of cement remains debated,^35^, ^36^, ^37^ although there is a trend toward an intermediate to high viscosity with the final say being that of the surgeon.^36^^,^^38^

Apart from ensuring appropriate cement viscosity, it is also recommended that excessive pressurization following cementation be avoided in those that are at high risk for developing BCIS. This includes patients with severe cardiopulmonary compromise, osteoporotic bone, pathologic hip and intertrochanteric fractures, an age >65 years old, an ASA score of three or over, femoral canals wider than 21 mm, underlying malignant tumors, bony or lung metastasis, and/or taking anticoagulants or diuretics.^16^^,^^34^^,^^36^ Other precautions in this procedure include promptly informing the anesthesia team during cement introduction and stem implantation, ensuring adequate intravenous hydration and urinary output, as well as the availability of vasopressors.

## 5 Conclusions

This systematic review and pooled analysis shows that long cemented femoral stems in hip arthroplasty for proximal femoral metastases are associated with significantly higher rates of perioperative cardiopulmonary complications compared to short or standard-length stems. Additionally, a very low incidence of new distal metastatic disease was found across all studies, calling into question the rationale behind usage of a longer stem. The majority of included studies were of moderate methodological quality, with only two meeting criteria for high quality, along with substantial heterogeneity in study design and reporting. While cemented fixation remains a reliable reconstructive strategy in this patient population, our findings demonstrate the need for new high quality studies to better assess the perioperative safety of using a long cemented stem in this setting. Future prospective comparative studies or randomized controlled trials should aim to use standardized definitions for perioperative cardiopulmonary complications and/or incorporate validated criteria for reporting BCIS, clearly define and attempt to standardize femoral stem length categories, and report the incidence of new distal metastatic lesions in the operated femur.

## CRediT authorship contribution statement

**Joseph D. Giacalone:** Conceptualization, Data curation, Formal analysis, Investigation, Writing – original draft, Writing – review & editing. **Jared Garfinkle:** Investigation, Methodology, Writing – original draft, Writing – review & editing. **Surabhi Panda:** Formal analysis, Methodology, Writing – review & editing. **Jason Abraham:** Visualization, Writing – review & editing. **Kassandra Parrales:** Validation, Resources, Writing – review & editing. **Christopher Haydel:** Supervision, Project administration, Writing – review & editing.

## Informed consent

Not applicable. This study did not involve human subjects or identifiable data.

## Guardian/patient's consent

Not applicable. This study did not involve active human participants, identifiable data, or minors requiring guardian consent.

## Institutional ethical committee approval

Not applicable. This study is a systematic review of previously published literature and did not require ethical approval.

## Ethical statement

This study did not involve human subjects or identifiable data and was therefore exempt from Institutional Review Board (IRB) approval.

## Funding/sponsorship

This research did not receive any specific grant from funding agencies in the public, commercial, or not-for-profit sectors.

## Conflict of interest

The authors declare no conflicts of interest.
